# Differences in the gut microbiome composition of Korean children and adult samples based on different DNA isolation kits

**DOI:** 10.1371/journal.pone.0264291

**Published:** 2022-03-10

**Authors:** Changyoon Baek, Woo Jin Kim, JaeWoo Moon, Seo Yoon Moon, Wonsub Kim, Hae-Jin Hu, Junhong Min

**Affiliations:** 1 School of Integrative Engineering, Chung-Ang University, Heukseok-dong, Dongjak-gu, Seoul, South Korea; 2 EOne Laboratories, Songdo, South Korea; 3 Endomics, Inc., Seongnam-si, Gyeonggi-do, Republic of Korea; Chung-Ang University College of Engineering, REPUBLIC OF KOREA

## Abstract

Recent studies have revealed that the composition of human gut microbiota varies according to region, race, age, diet, living environment, and sampling and DNA extraction method. The purpose of this study was to broaden our understanding of the intestinal microbial composition of Koreans by conducting a 16S rRNA amplicon sequencing on 78 Korean samples composed of adults, children, normal and obese groups. We compared the microbiome composition and diversity of these groups at different levels including the phylum and genus level using two different stool DNA extraction kits of QIAamp® PowerFecal® DNA Kit (Qiagen, Hilden, Germany) and CT Max Fecal DNA Kit (Ct bio, Korea). We found that Ct bio (Ct) kit recovered higher DNA yields and OTUs than QIAamp® PowerFecal® DNA Kit (Qia). The Ct kit, which adopted more rigorous bead beating method, detected the most Gram-positive (G+) bacteria, *Firmicutes*, at the Phylum level, whereas the Qia kit, which used a less rigorous cell lysis method, found the most Gram-negative (G-) bacteria, *Bacteroidetes*. The *Firmicutes*-to-*Bacteroidetes* (F/B) ratio showed no significant difference between the obese and the normal groups of same kit; however, they were significantly different with two different kits. There was a difference in the intestinal flora between healthy Korean adults and children. The taxa that differed significantly between the adults and children were *Bacteroides*, *Bifidobacterium*, *Prevotella*, and *Subdoligranulum*. There was no significant difference in the intestinal flora between the normal weight group and the obese group in adults and children, respectively. This is probably because the difference in body mass index (BMI) between the sample groups collected in this study is statistically significant, but it is not large enough to show a clear difference in the flora. Therefore, these results should be interpreted with caution while considering the BMI values and Korean obesity criterion together.

## Introduction

Microbiota in the human body plays a major role in the maintenance of human immune response, hormones, and homeostasis of the body [1]. Recently, studies have shown that not only obesity, diabetes, and liver disease, but medically demanding cancer and nervous system diseases also were related to the microbiome [2–5]. Microbiome research is expected to provide new opportunities for disease treatment and drug development [6].

Among the microbiome of the human body, the gut microbiota is of particular interest as it is known to regulate various host pathways. The composition of the intestinal microbiota, which is formed from infancy, is greatly affected by the method of childbirth and type of lactation and may vary depending on racial, environmental, and dietary factors [7–9]. Diet, microbiota, and host physiology and metabolism are interconnected, resulting in individual differences [10].

Recent studies have revealed that there is a close correlation between human disease and the microbiome, and thus, attempts are being made to apply it clinically. In addition, with the development of high-throughput DNA sequencing, diagnostic tests using this technology are increasing in hospitals and various clinical laboratories.

However, it is difficult to define a standard microbial community structure in a healthy person because the composition of the human microbiome varies by region, race, and living environment. In addition, it is difficult to evaluate the accuracy of the experiment as the microbiome study results depend on the sampling, extraction kit, analysis reagent, and data analysis method [11–14]. Therefore, selection of a kit that is suitable for the sample is expected to show excellent reproducibility, and a simple method of use will help maintain the quality of the test.

In this study, we tried to expand the understanding of intestinal microbial composition of Koreans by conducting a study with large number of samples and to help the study of diseases such as colorectal cancer or diabetes, which has rapidly increased among Koreans in recent decades. Korea had the second highest incidence rate of CRC in 2018 [15] and obesity is a major risk factor that may induce this. Also the prevalence of diabetes has increased mainly due to the increase in obesity in Korea [16].

We conducted a 16S rRNA amplicon sequencing on 78 Korean samples composed of adults, children, normal and obese groups and observed whether the differences in the composition of the intestinal flora appear consistently according to the DNA isolation kit. For this purpose, two stool DNA extraction kits were applied: QIAamp^®^ PowerFecal^®^ DNA Kit (Qiagen, Hilden, Germany) and CT Max Fecal DNA Kit (Ct bio, Korea). QIAamp^®^ PowerFecal^®^ DNA Kit (Qia kit) is a commonly used commercial product that uses heat and bead beating for cell lysis. Whereas CT Max Fecal DNA Kit (Ct kit) uses rigorous bead beating method at ambient temperature.

We also observed whether there is a difference in the intestinal flora between healthy Korean adults and children and between the normal weight and obese groups of adults and children respectively. We hope that the results of this study will be used as reference data for research on obesity-related diseases in Koreans.

## Results

### Subject characteristics

A total of 78 samples, 49 adults and 29 children, were included in our 16S rRNA data analysis (Table 1). Each group was further divided into normal and obese groups according to body mass index (BMI) status (Methods).

**Table 1:** Demographic characteristics of the study population.

	Normal Children (n = 18)	Obese Children (n = 11)	*P*	Normal adults (n = 21)	Obese adults (n = 28)	*P*
Age (yr) (range)	8.7±3.3 (3–14)	11.2±3.3 (6–17)	0.06*	47.1±11.4 (22–63)	46.4±10.8 (25–62)	0.823
Gender (M/F)	7/11	8/3	0.388^†^	2/19	13/15	**0.011**
Height (cm)	134.9±22.4	150.36±14.1	0.05	160.3±7.8	166.4±11.5	**0.037**
Height (Z-score)	0.29±0.8	0.48±1.2	0.612	NA	NA	NA
Weight (kg)	30.1±10.1	57.1±12.3	**<0.00001**	51.1±7.5	74.3±11.2	**<0.00001**
Weight (Z-score)	-0.44±0.9	1.7±0.9	**<0.00001**	NA	NA	NA
BMI (kg/m2)	16.2±2.2	25.0±2.1	**<0.00001**	19.8±1.2	26.8±2.5	**<0.00001**
BMI (Z-score)	-0.73±1.3	2.0±0.9	**<0.00001**	NA	NA	NA

NA: not applicable.

Values are presented as mean±SD.

BMI z-score: Z-score-converted value from the 2017 Korean growth chart.

*Student’s t-test was used to compare the mean values for age, height, weight and BMI.

^†^Fisher-exact test was used to compare the group. Bold letters signify *p* < 0.05.

There was no difference between the age of adults in both the normal and the obese group. In the children group, the age of the obese children was slightly higher than that of the normal children, but the difference was not statistically significant.

Gender of the children group was similarly distributed in the normal and obese groups. In the adult obese group, the gender ratio was similar, while the normal group comprised mostly female participants.

The children in the obese group were slightly taller than their normal counterparts, but the difference did not seem to be significant. In the adult group, the average height of obese adults was slightly higher than that of normal adults.

There was a difference in weight and BMI between the normal and obese group in both the adult and children groups.

### DNA yield and purity

**Fig 1:**
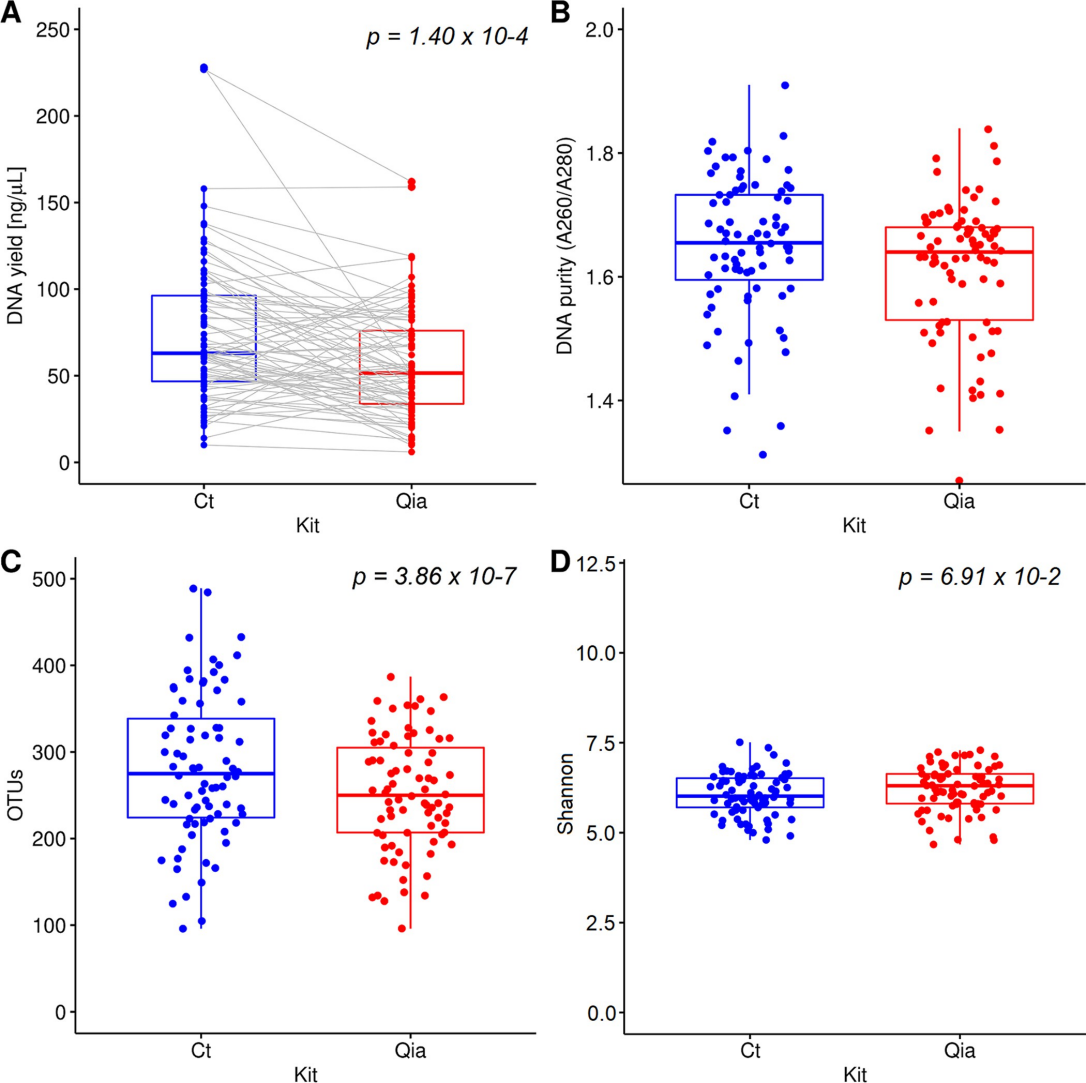
Comparison of DNA quality and bacterial diversity using two DNA isolation kits (Ct and Qia). A. DNA yield. The gray lines represent the same sample pair. B. DNA purity. C. OTUs D. Chao1 index.

Higher DNA yields were obtained with the Ct isolation kit (*p* = 1.4 x 10^−4^) (Fig 1A). DNA purity (A260/A280 ratio) was within the expected range (1.6–2.0) for about 76% of the samples for Ct and 71% for Qia kit (Fig 1B).

### Community richness and diversity of all samples and subgroups

Seventy-eight stool samples were sequenced after DNA extraction using Ct and Qia isolation kits. After filtering out low-quality and chimeric sequences, we obtained a total of 1,818,276 (range, 15,054–31,517) and 1,072,362 (range, 5,296–28,707) high-quality reads from Ct and Qia kit, respectively. Each sample was covered by an average of 23,311 (Ct) and 13,748 reads (Qia).

The bacterial diversity was compared using the number of observed Operational Taxonomic Units (OTUs) and the Shannon’s diversity metric (Fig 1C and 1D). The Ct kit showed more OTUs than Qia kit (*p* = 3.86 x 10^−7^). The median OTUs were 275 (range, 96–489) and 250 (range, 96–387) for Ct and Qia, respectively. Since the Ct kit applies more rigorous mechanical lysis for DNA isolation, it may have higher DNA yield and OTUs compared to those obtained for Qia.

When evaluated the Shannon’s diversity which considers not only the number of species but also the evenness of their abundance, there was no significant difference between the Qia and the Ct kit (*p* = 0.0691). The Qia kit with both chemical and mechanical lysis appears to have colony uniformity similar to the Ct kit with rigorous bead beating lysis.

When the adult and the children group (49 and 29 each) and the normal and obese groups (39 each) were compared, there was no significant difference found in the colony richness and diversity for both isolation kits (S1 Table).

### Comparison of fecal microbial diversity and composition

**Fig 2:**
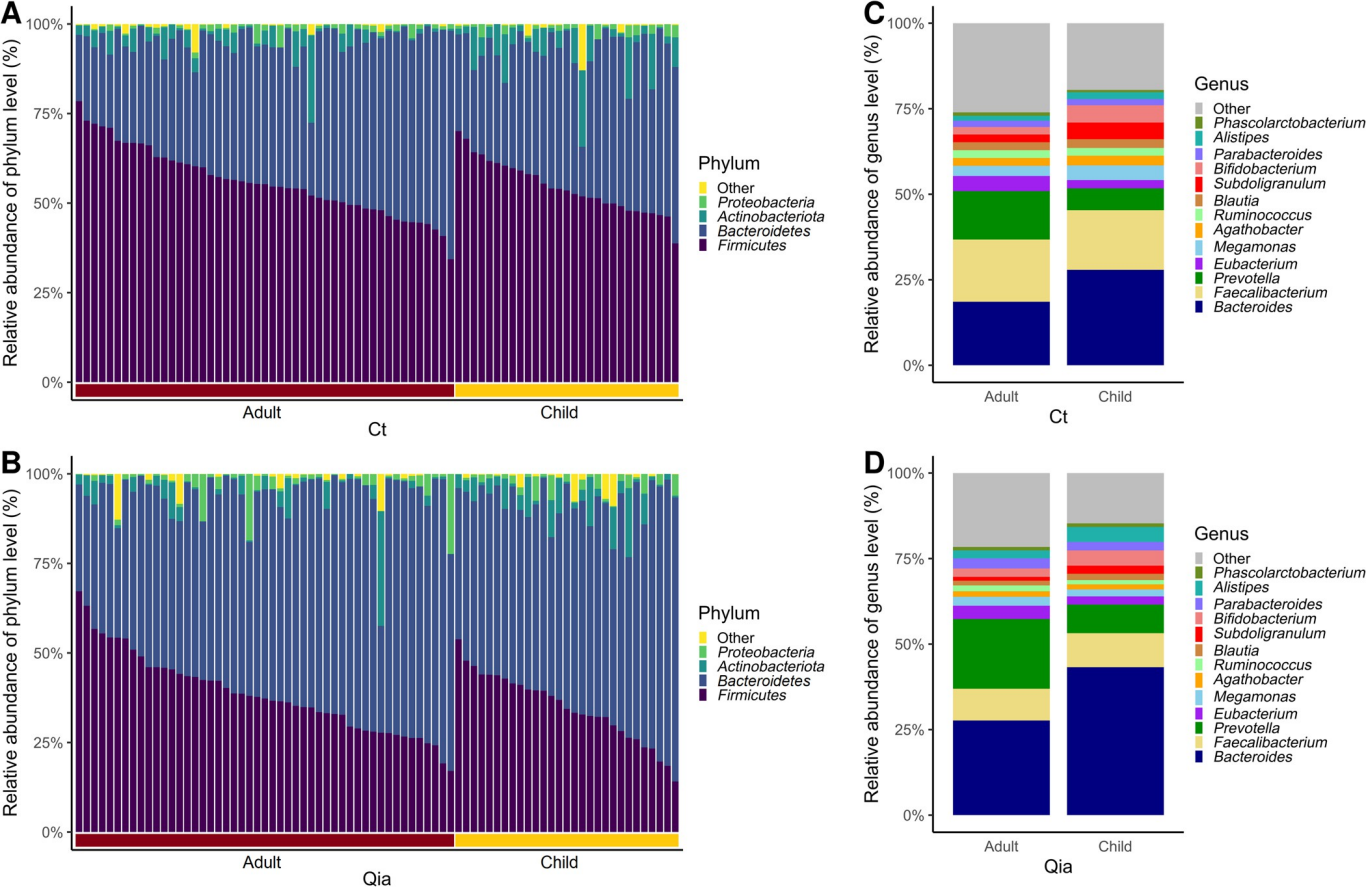
Relative abundance of microbiome of adults and children. A. Variation in bacterial relative abundance at the phylum level with Ct kit. B. Bacterial relative abundance at the phylum level with Qia kit. C. Bacterial relative abundance at the genus level with Ct kit. D. Bacterial relative abundance at the genus level with Qia kit.

At the phylum level, four phyla, *Bacteroidetes; Firmicutes; Actinobacteriota; and Proteobacteria*, showed relative abundance exceeding the filtering criteria (≥ 1% in at least 10% of total samples) in both isolation kits (Fig 2A and 2B).

**Fig 3:**
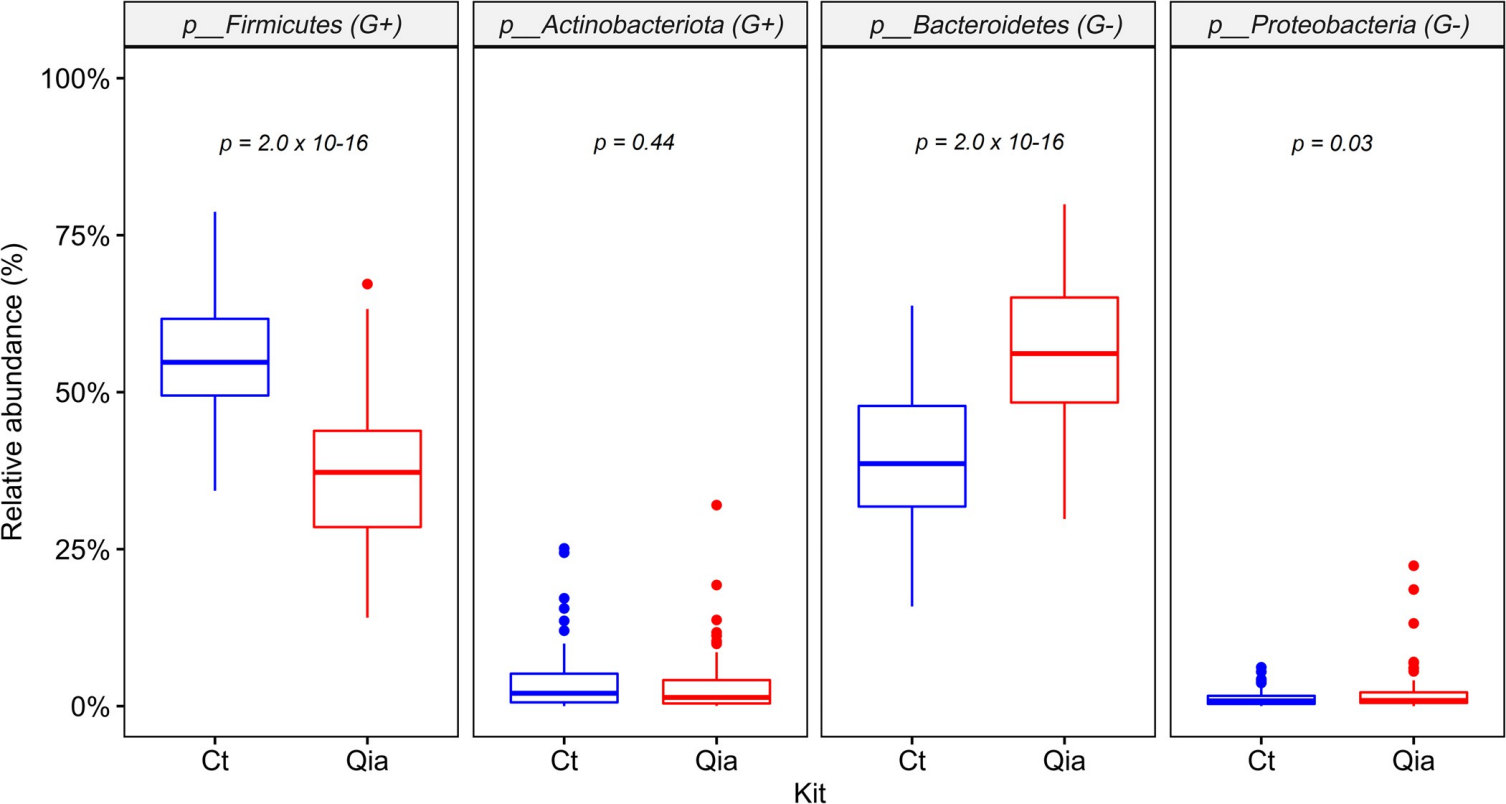
Comparison of relative abundance of four major microbiomes at phylum level using Ct and Qia kits. G+: Gram-positive bacteria. G-: Gram-negative bacteria.

Interestingly, *Firmicutes*, a Gram-positive (G+) bacteria was the most abundant in the Ct kit, followed by a Gram-negative (G-) *Bacteroidetes*. Conversely, in the Qia kit, *Bacteroidetes* was the most abundant, followed by *Firmicutes*. (Figs 2A, 2B and 3). When the relative abundance of these two phyla was observed in both kits for the same sample, Ct kit showed approximately 17% lower abundance of *Bacteroidetes* and approximately 19% higher abundance of *Firmicutes*, on an average, than the Qia kit (S1 Fig).

This trend was also observed in case of other phyla, *Actinobacteriota* and *Proteobacteria*, although the differences were small: *Actinobacteriota* (G+) was slightly higher in the Ct kit (median, 2.05% vs. 1.39%), while *Proteobacteria* (G-) was slightly higher in the Qia kit (median 0.84% vs. 0.90%) (Fig 3).

**Fig 4:**
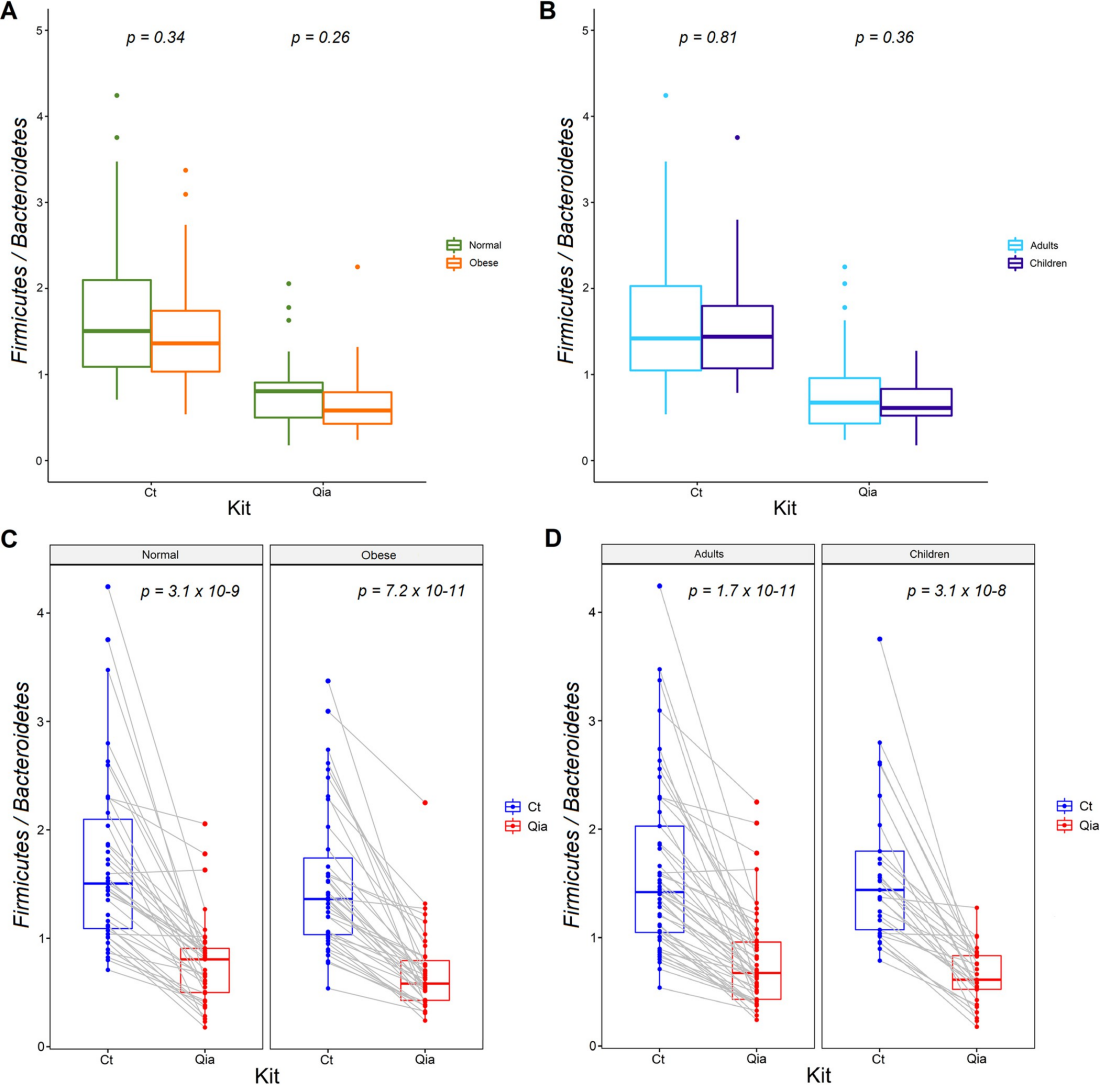
*Firmicutes*/*Bacteroidetes* (F/B) ratio of subgroups. A. F/B ratio distribution between normal vs. obese group. B. F/B ratio distribution between adult vs. children group. C. F/B ratio distribution between Ct and Qia kits in Normal and obese groups, respectively. The gray lines represent the same sample pair. D. F/B ratio distribution between Ct and Qia kits in adults and children groups, respectively.

We divided all samples into obese and normal groups and observed the *Firmicutes*-to-*Bacteroidetes* (F/B) ratio. There was no difference in F/B ratio between the two groups (Fig 4A), which is consistent with our previous study [17]. The ratio did not differ between the adults and children groups either (Fig 4B). Although the F/B ratios were not different between the two subgroups, the values were significantly different between the two kits (Fig 4C and 4D). Median values of F/B ratio in the normal group were 1.50 and 0.81, obese group were 1.36 and 0.58, adult group were 1.42 and 0.67, and the children group were 1.44 and 0.61 for the Ct and the Qia kits, respectively.

The non-parametric Mann-Whitney U test identified that *Actinobacteriota* tended to be more abundant in the children than in the adult group at the phylum level. This difference appears to be more significant with Qia than the Ct kit (FDR-adjusted *p* = 0.0078 vs. *p* = 0.0516, respectively). Interestingly, *Actinobacteriota* was more abundant in normal-weight children than in normal-weight adults in the Qia and the Ct kits (FDR-adjusted *p* = 0.0253 vs. *p* = 0.0968, respectively), while no difference was observed between the obese adults and the obese children groups.

**Fig 5:**
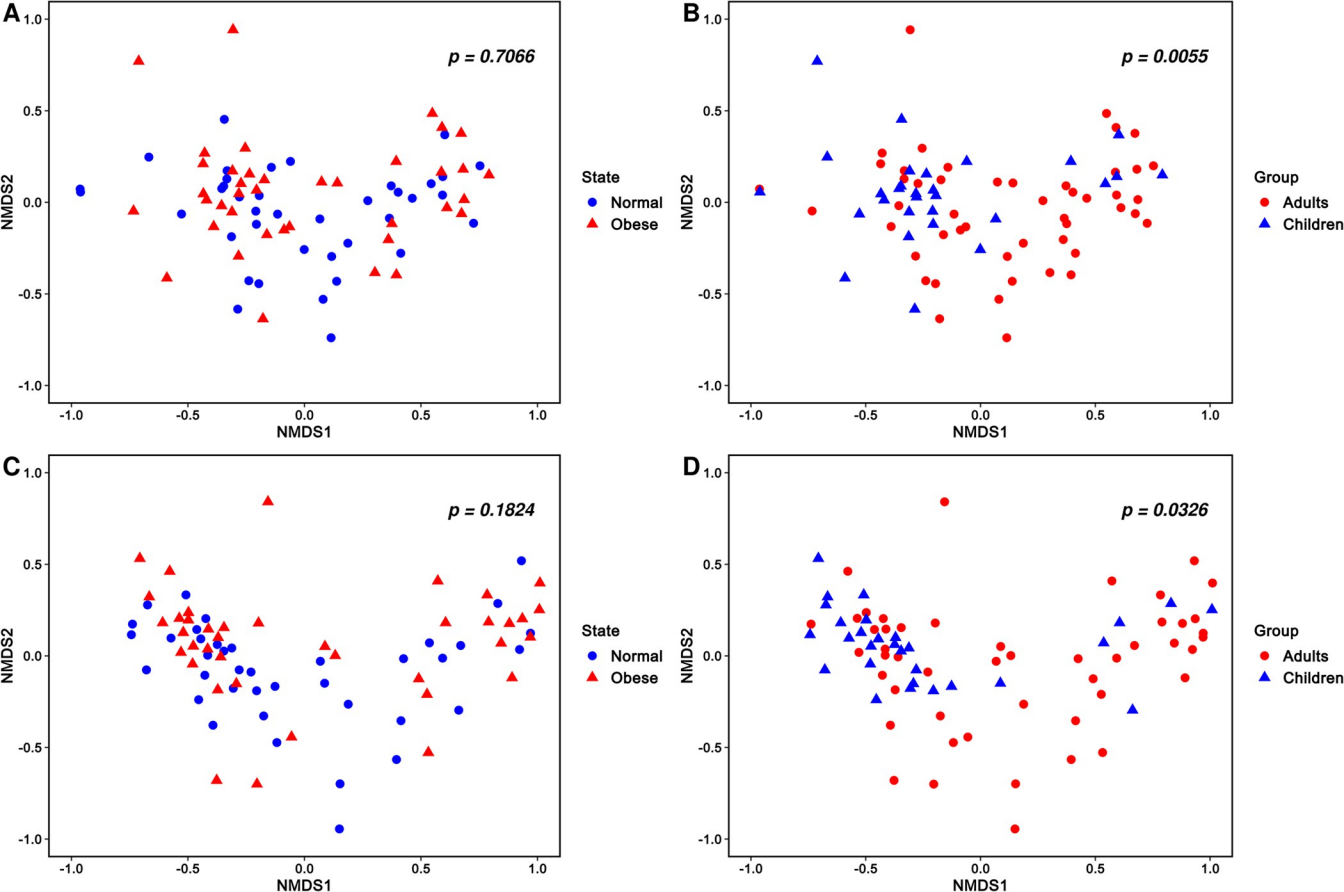
Beta diversity observation in different subgroups. A. NMDS plot of normal and obese group microbiome communities with Ct kit. B. NMDS plot of adults and children microbiome communities with Ct kit. C. NMDS plot of normal and obese group microbiome communities with Qia kit. D. NMDS plot of adults and children microbiome communities with Qia kit.

When observed at the genus level, adult and children groups differed significantly in bacterial diversity, whereas there was no difference between the normal and obese groups (Fig 5). In Fig 5, Bray-Curtis distance metric was used. However, consistent results were obtained for both the Ct and the Qia isolation kits in other metrics including Unweighted unifrac, Weighted unifrac, and Jaccard distance. For the same individual, the genus-level relative abundance of major taxa was highly correlated (n = 78 paired samples, average Pearson *r* = 0.7).

The taxa that differed significantly between the children and adults were *Bacteroides*, *Bifidobacterium*, *Prevotella*, and *Subdoligranulum* for both the kits (Table 2, Fig 2). *Bacteroides*, *Bifidobacterium*, and *Subdoligranulum* were more abundant in children, whereas *Prevotella* was enriched in the adults. This observation was similar in the major taxa between the normal children and adults and between the obese children and adults in both kits (S2 Fig).

**Table 2:** List of taxa showing different abundance between adults and children at the genus level.

Genus	Ct kit				Qia kit			
	P-value* (FDR adjusted)	Adults (mean)	Children (mean)	Fold change (Adults/ Children)	P-value* (FDR adjusted)	Adults (mean)	Children (mean)	Fold change (Adults/ Children)
*Agathobacter*	0.9053	0.023	0.029	0.82	0.6645	0.016	0.015	1.06
*Alistipes*	0.2228	0.014	0.020	0.69	**0.0282**	0.023	0.043	0.54
*Bacteroides*	**0.0141** †	0.186	0.279	0.67	**0.0061**	0.277	0.433	0.64
*Bifidobacterium*	**0.0141**	0.022	0.050	0.44	**0.0061**	0.025	0.045	0.55
*Blautia*	0.0529	0.023	0.025	0.90	0.2270	0.014	0.019	0.73
*Eubacterium*	**0.0093**	0.044	0.024	1.86	0.0674	0.039	0.024	1.64
*Faecalibacterium*	0.7696	0.182	0.174	1.04	0.7941	0.092	0.099	0.93
*Megamonas*	0.4499	0.029	0.043	0.69	0.8969	0.026	0.020	1.29
*Parabacteroides*	0.1643	0.019	0.018	1.03	0.2791	0.030	0.025	1.18
*Prevotella*	**0.0016**	0.141	0.064	2.21	**0.0167**	0.204	0.084	2.43
*Ruminococcus*	0.8114	0.023	0.023	0.99	0.7941	0.018	0.013	1.39
*Subdoligranulum*	**0.0078**	0.023	0.049	0.47	**0.0282**	0.011	0.023	0.47

*Mann-Whitney U test.

†FDR-adjusted significant *p*-values are marked in bold.

**Fig 6:**
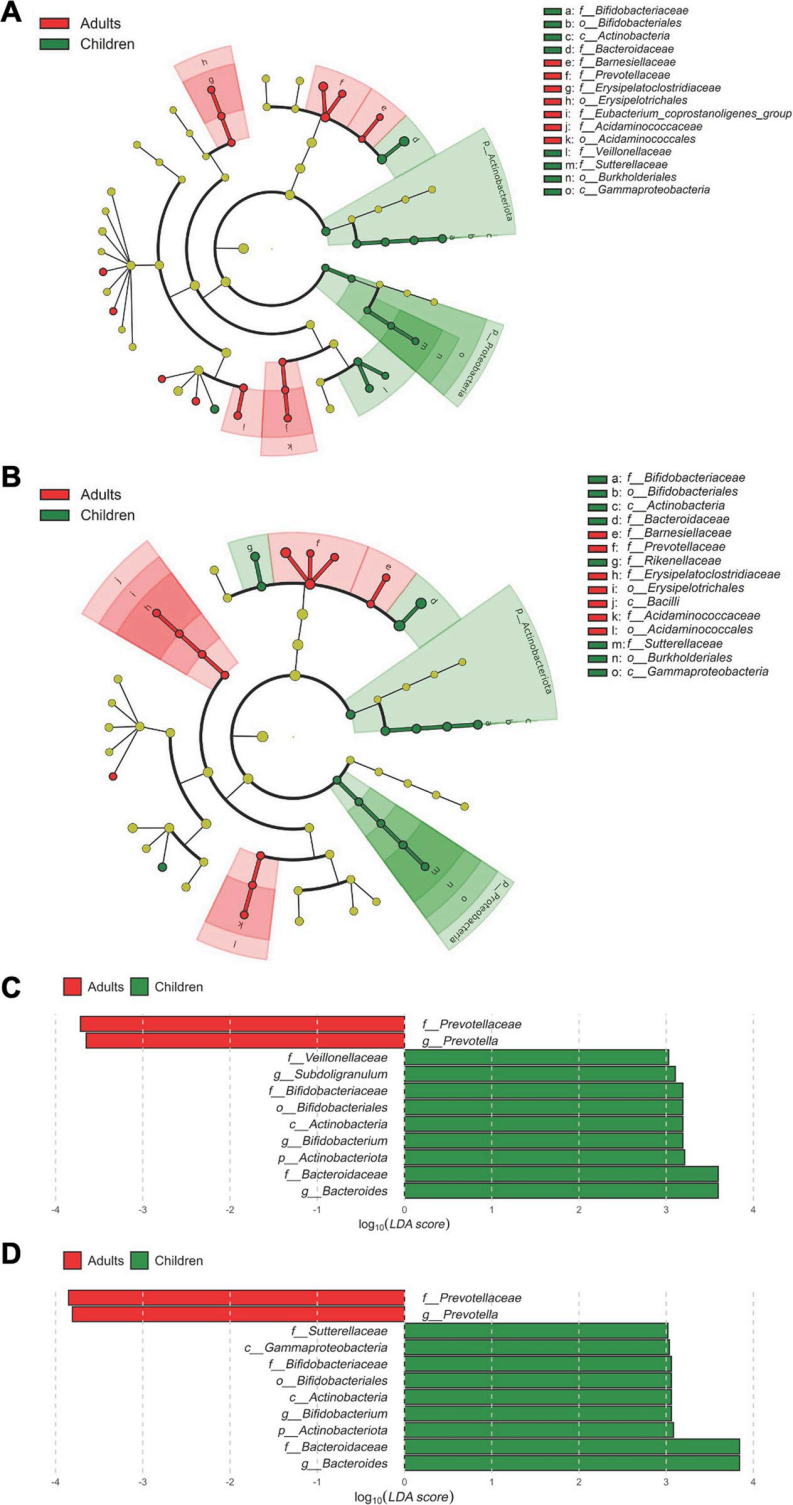
Characterization of microbiomes of adults and children by LEfSe and LDA analysis. A. Taxa with significant differences in abundance between adults and children with Ct kit. B. Taxa with significant differences in abundance between adults and children with Qia kit. Histogram of log_10_(LDA scores) for features with differential abundance between adults and children with Ct kit (C) and with Qia kit (D). Taxa of |log_10_(LDA scores)| >3 are presented.

We performed LEfSe (Linear discriminant analysis effect size) to identify bacterial taxa that could best explain the differences between adults and children with a logarithmic cutoff value of linear discriminant analysis (LDA) > 2.0. The results showed that *Actinobacteriota* and *Proteobacteria* were enriched in children than in adults and was commonly observed in both the Ct and Qia kits (Fig 6). The histogram of the LDA scores indicated that *Bacteroides*, *Actinobacteriota*, and *Bifidobacterium* were enriched in children, while *Prevotellaceae* was more abundant in adults. This is consistent with the relative abundance of genus-level taxa.

## Methods

### Sample collection

Stool samples were collected from 78 participants. A fresh stool sample (~ 200 mg) was placed into a collection container with dry ice and brought to the study center within 12 h. The collected stool samples were stored in tubes at -20°C prior to DNA extraction.

All experiments on stool samples were approved by the ethics committee of Cheju Halla General Hospital (CHHIRB-2015-L06-01). Informed consents were obtained from human participants of this study.

According to the obesity treatment guidelines of the Korean Society for Obesity in 2018, a BMI of 23 or higher was defined as overweight, and a BMI of 25 or higher as obese for adults. Likewise, according to the 2017 growth chart for children and adolescents, BMI between 21.6 and 23.5 was defined as overweight, and BMI > 23.5 as obese. The BMI Z-score of child was calculated using the following LMS formula [18] along with the 2007 Korean National Growth Chart:

$$Z=\left({\left(\frac{x}{M}\right)}^{L}-1\right)/LS,\;L\neq 0$$ (1)

$$Z=\log\left(\frac{x}{M}\right)/S,\;L=0$$ (2)

where, x is height, weight or BMI value; and the L, M, and S data for each child by gender and age are available on the Korea Centers for Disease Control and Prevention website (https://knhanes.kdca.go.kr).

### DNA extraction

DNA was extracted using two isolation kits, QIAamp® PowerFecal® DNA Kit (Qiagen, Hilden, Germany) and CT Max Fecal DNA Kit (Ct bio, Seoul, Korea).

DNA extraction using Qia kit was performed according to the manufacturer’s instructions without modification. Briefly, the protocol involved homogenizing the stool sample by using a heat treatment at 65°C for 10 min and a bead beating method (0.7 mm diameter; included in the kit) for 10 min at 50 Hz, followed by aggregation using trivalent cations such as Al^3+^, and finally precipitation by centrifugation to remove impurities. This protocol required a low temperature (4°C) to improve the aggregation efficiency. The entire process took approximately 40 min.

DNA extraction using CT Max Fecal DNA Kit (Ct) was performed as follows: The stool sample (~200 mg) was loaded in the first tube, in which 0.4 g of glass micro beads (0.07~0.1 mm diameter) and M1 buffer were included. In the first tube, the stool was broken up, and bacteria were simultaneously released and lysed by bead beating (50 Hz) for 1 min. Big stool debris was settled to the bottom of the first tube by centrifugation (4,300 x g, 1 min). The supernatant was transferred to a second tube with M2 buffer and was briefly mixed and centrifuged to separate small stool debris into the supernatant. The lower part of the solution was mixed with M3 buffer and loaded to a binding column. For normal DNA recovery, the M3 buffer was discarded from the binding column, with the DNA adsorbed to the binding column. The binding column in the second tube was washed 2 times with M4 and M5 buffer and finally, the adsorbed DNA was eluted using the M6 buffer. The entire process took approximately 15 min at room temperature (25°C), and no separate heating or cooling procedures were required.

### Evaluation of DNA yield and purity

The DNA yield was estimated by the Picogreen method (Invitrogen, USA) using Victor 3 fluorometry (Perkin-Elmer, USA). The extracted DNA concentration, and the 260/280 absorbance ratio, which is defined as purity of DNA, was measured using Nano Drop ND-1000 spectrophotometer (Nanodrop Technologies Inc., NC, USA).

### Library construction and sequencing

The sequencing libraries were prepared according to the Illumina 16S Metagenomic Sequencing Library protocols to amplify the V3 and V4 regions.

The 2 ng of input gDNA was PCR amplified with 5x reaction buffer, 1 mM of dNTP mix, 500 nM each of universal F/R PCR primers, and Herculase II fusion DNA polymerase (Agilent Technologies, Santa Clara, CA). The conditions for PCR were: 3 min at 95°C for heat activation, and 25 cycles of 30 sec at 95°C, 30 sec at 55°C and 30 sec at 72°C, followed by a 5-min final extension at 72°C. The universal primer pair with Illumina adapter overhang sequences were as follows:

V3-F: 5’-TCG TCG GCA GCG TCA GAT GTG TAT AAG AGA CAG CCT ACG GGN GGC WGC AG-3’,

V4-R: 5’- GTC TCG TGG GCT CGG AGA TGT GTA TAA GAG ACA GGA CTA CHV GGG TAT CTA ATC C-3’.

The PCR product was purified with AMPure beads (Agencourt Bioscience, Beverly, MA). Following purification, 2 μl of the PCR product was PCR amplified the second time for final library construction containing the index using NexteraXT Indexed Primer. The cycle condition for 2^nd^ PCR was same as that for the 1^st^ PCR except for 10 cycles. The PCR product was purified with AMPure beads.

The final purified product was then quantified using qPCR according to the qPCR Quantification Protocol Guide (KAPA Library Quantification kits for Illumina Sequencing platforms) and qualified using the TapeStation D1000 ScreenTape (Agilent Technologies, Waldbronn, Germany).

The paired-end (2×300 bp) sequencing was performed by the Macrogen (South Korea) using the MiSeq™ platform (Illumina, San Diego, USA).

### Sequencing data analysis

Forward and reverse paired end 16S rRNA sequences were merged using the tool QIIME 2 (version: 2020.8.0). The merged sequences were demultiplexed and divided into samples using the barcode sequence of each sample. Using QIIME 2 plugin Deblur, quality control was performed, and the noise was removed, resulting in obtention of high-quality sequence data.

Alpha and beta diversity analysis was performed using the diversity plugin of QIIME 2. Non-metric Multi-dimensional Scaling (NMDS) plots were drawn using R packages “vegan” and “ggplot2”.

Taxonomy was assigned to the sequences (Operational Taxonomic Unit, OTUs) using a Naive Bayes classifier pre-trained on Silva reference database (silva-138-99-nb-classifier) and the feature-classifier plugin of QIIME 2. The compositional microbiome data were computed on seven taxonomic levels (species, genus, family, order, class, phylum, and kingdom). For each sample, taxa with a relative frequency more than or equal to 1% in at least 10% of the total sample were selected for analysis. Taxa without annotations down to the genus level and those beginning with a simple "UCG-" were excluded.

Linear Discriminant Analysis (LDA) Effect Size (LEfSe) method was applied to analyze the differences in bacterial abundance between groups at different taxonomic levels using default parameters [19]. Two groups were considered significantly different at *p* value < 0.05 and | log_10_(LDA score) | > 2.

### Statistical analyses

Statistical analyses were performed with the R package (version 3.4.4, https://www.r-project.org/) and QIMME 2 plugins. Fisher’s exact test was performed to check whether gender showed an equal distribution between groups. Age, height, weight and BMI were compared using Student’s t-test. Alpha diversity indices were measured from the Krusal-Wallis test (QIIME 2) and Mann-Whitney U test. Beta diversity was measured from the pairwise PERMANOVA (Permutational multivariate analysis of variance) test using Bray-Curtis, Unweighted unifrac, Weighted unifrac, and Jaccard distance metrices. Non-parametric Mann-Whitney U test was performed for the comparison of microbial composition between the adults and children and normal and obese groups on each taxonomy level. FDR multiple test correction was performed with the R package. The resulting *p*-values were adjusted for multiple hypothesis testing using FDR correction, and results were considered significant at FDR = 5%.

## Discussion

One of the challenging aspects of human microbiome research is the differences incurred by region, race, and living environment. This makes it difficult to define a standard microbial community structure for a healthy person. Large-scale studies have been conducted to characterize the microbiome of healthy people in various countries. In this study, we tried to expand the understanding of the composition of intestinal microbes in Koreans by conducting a study with large number of Korean samples, and to help the study of diseases such as colorectal cancer or diabetes, which has rapidly increased among Koreans in recent decades.

Another challenging aspect is that the bacterial composition may vary depending on the sampling kit or DNA extraction method [12–14]. The various lysis procedures, such as enzymatic, chemical or mechanical methods of the commercial extraction kits, are presumed to have a significant impact on bacterial composition. In this study, we evaluated the microbial composition and diversity in two Korean sample groups (adults vs children, and normal vs obese) using two different DNA extraction kits. DNA was isolated using two kits for each of the 78 subjects, to determine the effect of the DNA isolation method on the microbial composition.

The Ct kit showed higher DNA yield and OTUs than Qia kit. This difference is presumed to be related to the lysis efficacy of the kit. In the Qia DNA extraction kit, both chemical and mechanical lysis were used with 0.7 mm diameter beads, whereas, in the Ct kit, only mechanical lysis is applied with 0.07 to 0.1 mm diameter beads. We consider that the smaller bead diameter used in the Ct kit were more effective for cell lysis than those in the Qia kit, consistent with reports in a recent study [20].

Several previous studies have reported that bead beating method is critical to improve the lysis of Gram-positive bacteria and obtain a high DNA concentration [11, 21, 22], which was consistent with our results. In the Ct kit, which extracted DNA through more rigorous bead beating, *Firmicutes* (G+) was most abundant, followed by *Bacteroidetes* (G-) at the phylum level. Conversely, in the Qia kit, *Bacteroidetes* (G-) was most abundant, followed by *Firmicutes* (G+).

F/B ratio did not differ between the normal and the obese groups in both kits. This is consistent with our previous study on normal and obese Korean children [17]. However, the F/B values between the two DNA isolation kits were significantly different. The median F/B values of the normal versus obese group of the Ct kit were 1.5 vs. 1.36 and those for the Qia kit were 0.81 vs. 0.58. Since there are colonies that are better or less detectable depending on the DNA isolation kit, caution should be exercised when comparing F/B values from studies derived using different DNA extraction kits.

In this study, we observed that *Actinobacteriota* was more abundant in children than in adults in both kits, which is contradictory to a previous study [23] reporting more abundance in adults than children. According to the study of Hildebrand et al. [24], *Actinobacteriota* peaks at the age of 15, decreases with age and tends to rise again at the age of 48. Their results indicate that the abundance of this taxon may be observed differently depending on the age groups selected as adults and children.

The microbial composition between the adult and children groups in this study at the genus level were both in agreement and contradiction with other studies. The higher abundance of *Bifidobacterium* in children than in adults is in accordance with other studies [25, 26]. *Bacteroidetes* were more abundant in children than adults in our study, consistent with studies of Derrien et al. [26] and Radjabzadeh et al. [27] and contradicting that of Hollister et al [25]; in our study, *Prevotella* was more abundant in the adults, whereas Derrien et al. reported more abundance in children. This inconsistency was presumed to be dependent on race, age, genetics, environmental, technical, and clinical factors.

In this study, there was no significant difference in the microbial composition between the normal weight and the obese group in adults and children, respectively, in both kits. This is probably because the difference in BMI between the sample groups collected in this study is statistically significant, but it is not large enough to show a clear difference in the flora. The average BMIs of the normal and obese children group was 16.2 and 25.0, respectively, and the average BMIs of the normal and obese adult group was 19.8 and 26.8, respectively (Table 1). Considering that Korean children with a BMI of 23.5 or higher and Korean adults with a BMI of 25 or higher are classified as obese, the average BMI value of the obese adults and children group is just above the obesity criterion. Therefore, these results should be interpreted with caution while considering the BMI values and Korean obesity criterion together.

The purpose of this study was to observe whether the composition of the intestinal flora differed between adults and children, and between normal and obese individuals, using two different DNA extraction kits. Although the number of samples in each group was not large enough (49 samples for adults, 29 samples for children, 39 samples for normal and 39 samples for obese), the results observed in the two different DNA isolation kits were consistent between groups.

A limitation of this study is that dietary information and biochemical markers such as glucose, triglycerides, total cholesterol could not be collated at the time of sample collection, which will be addressed in future research.

In summary, we confirmed the difference in the gut microbiome of Korean children and adult samples using two different DNA isolation kits. No significant difference was observed in the intestinal flora between the normal group and the obese group in this study, where the difference in BMI was not sufficiently large. Consistent with other studies, there were differences in bacterial composition depending on the DNA extraction kit, but both kits showed consistent results when comparing groups within the same kit.

## Supporting information

S1 Table Comparison of gut community richness and diversity between adults and children, and normal and obese groups. Values are presented as *p*-values. Bold letters indicate significant differences (Mann-Whitney U-test, p < 0.05).
(DOCX)

S1 Fig Comparison of relative abundance of *Bacteroidetes* and *Firmicutes* in Ct and Qia kits for the same sample. A. *Bacteroidetes*. B. *Firmicutes*.
(TIF)

S2 Fig Relative abundance of subgroup microbiome at genus level. A. Normal weight group: adults vs. children, left: Ct kit, right: Qia kit. B. Obese group: adults vs. children, left: Ct kit, right: Qia kit.
(TIFF)
