# Supplementary material for: Differences in the gut microbiome composition of Korean children and adult samples based on different DNA isolation kits
Source: PLoS One. 2022 Mar 10;17(3):e0264291. doi: 10.1371/journal.pone.0264291 (PMC8912269; doi:10.1371/journal.pone.0264291)
Supplement: S1 Table — Values are presented as p-values. Bold letters indicate significant differences (Mann-Whitney U-test, p < 0.05). (DOCX) [file pone.0264291.s001.docx]

|  | Dominance | |  | Evenness | |
| --- | --- | --- | --- | --- | --- |
|  | Ct kit | Qia kit |  | Ct kit | Qia kit |
| Adults vs. children* |  |  | Adults vs. children* |  |  |
| Mcintosh_d | 0.5522 | 0.6982 | Simpson | 0.4724 | 0.5591 |
| Simpson | 0.5522 | 0.7213 | Shannon | 0.4598 | 0.8972 |
| Normal vs. Obese† |  |  | Normal vs. Obese† |  |  |
| Mcintosh_d | 0.0982 | 0.0884 | Simpson | **0.0162** | 0.0942 |
| Simpson | 0.0982 | 0.0961 | Shannon | 0.1109 | **0.0181** |

*n = 49 adults and 29 children

†n = 39 normal and 39 obese
