# Supplementary figures and images for: Differences in the gut microbiome composition of Korean children and adult samples based on different DNA isolation kits
Source: PLoS One. 2022 Mar 10;17(3):e0264291. doi: 10.1371/journal.pone.0264291 (PMC8912269; doi:10.1371/journal.pone.0264291)

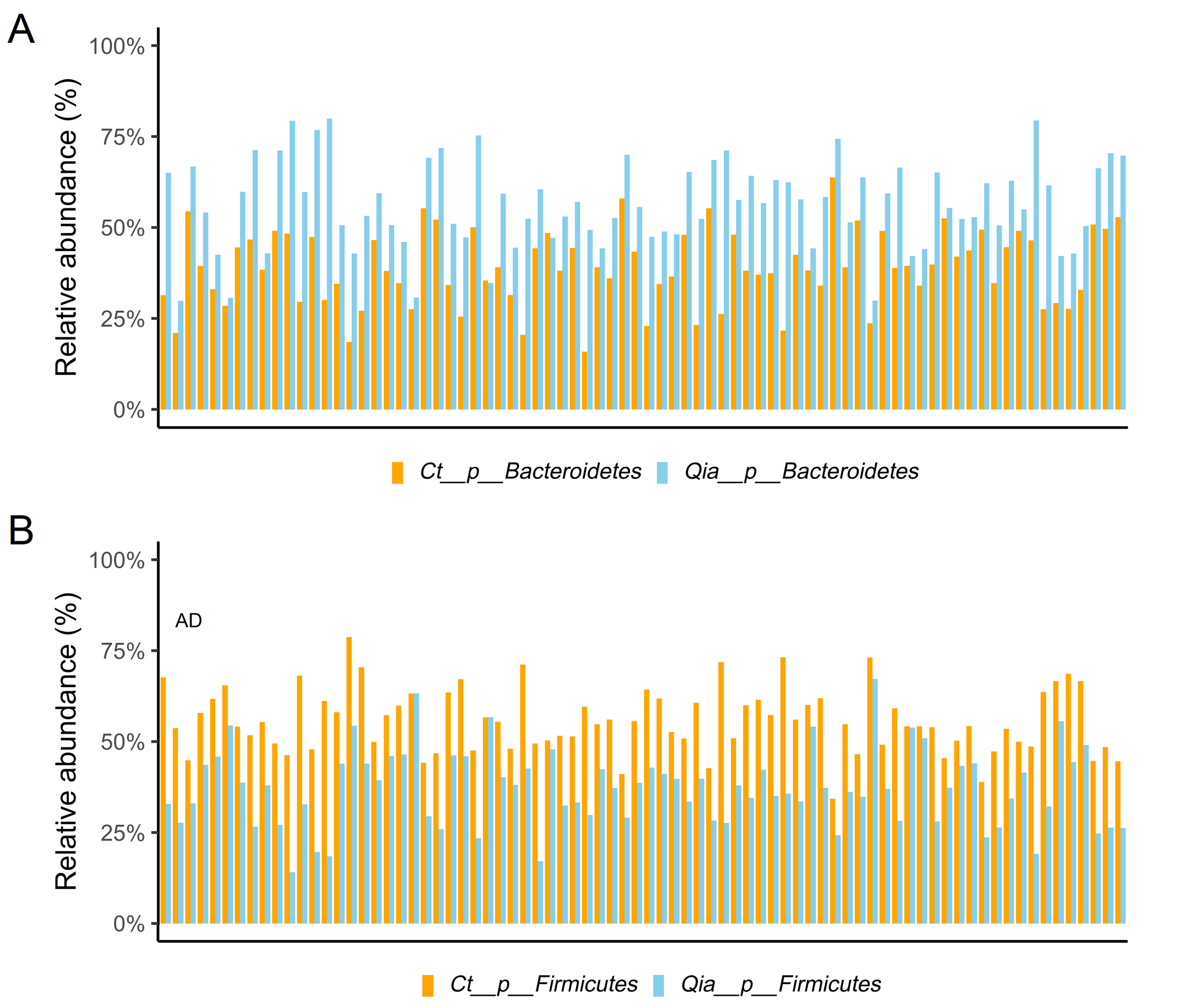

Supplement: S1 Fig — A. Bacteroidetes. B. Firmicutes. (TIF) [file pone.0264291.s002.tif]

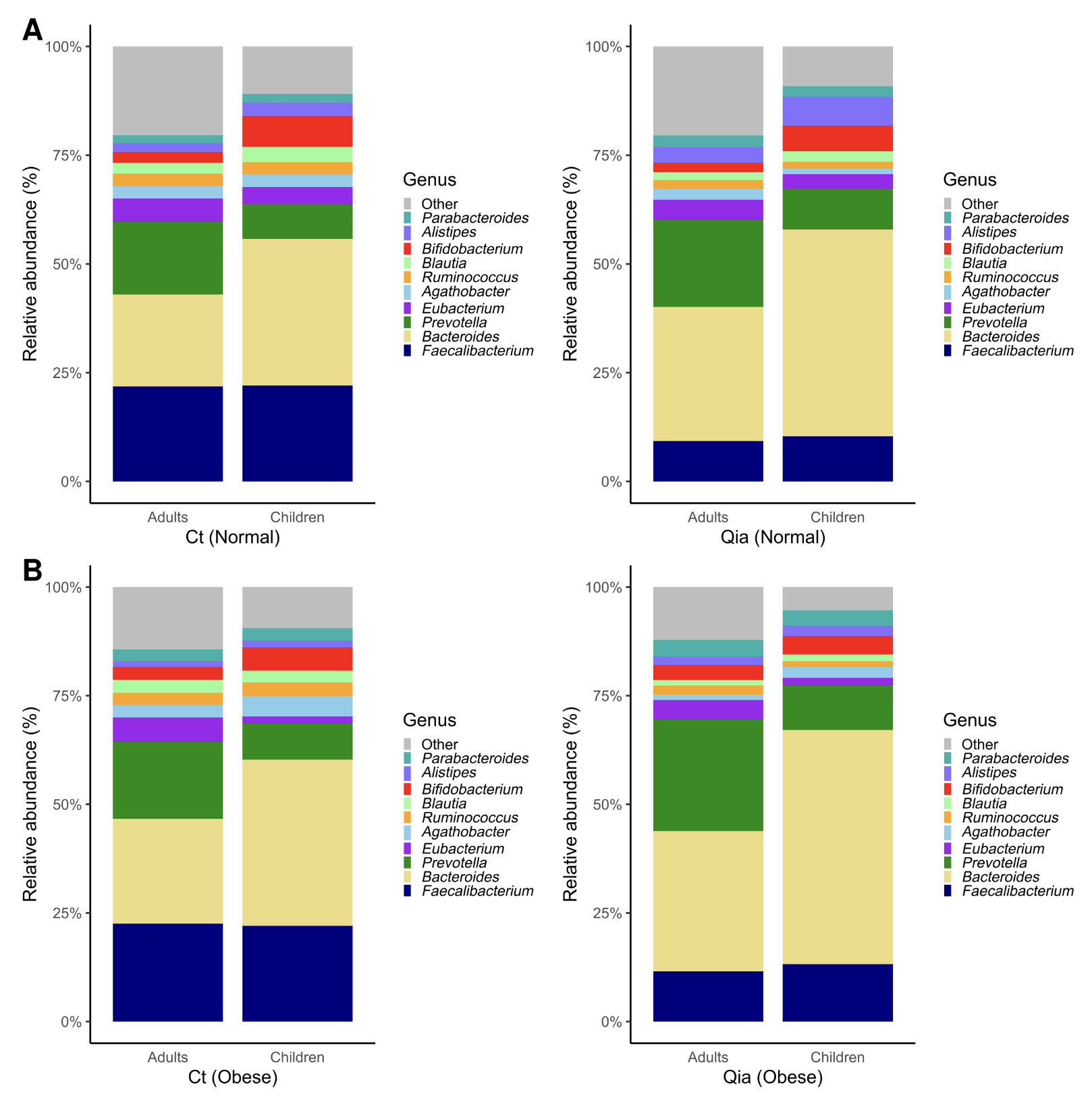

Supplement: S2 Fig — A. Normal weight group: adults vs. children, left: Ct kit, right: Qia kit. B. Obese group: adults vs. children, left: Ct kit, right: Qia kit. (TIFF) [file pone.0264291.s003.tiff]
